# Hemodiafiltration: a synergy yet to be convincing

**DOI:** 10.1590/2175-8239-JBN-2024-PO02en

**Published:** 2024-03-25

**Authors:** Brammah Rajarajeswaran Thangarajah

**Affiliations:** 1 University of Jaffna, Department of Medicine, Jaffna, Sri Lanka.

**Keywords:** Renal Dialysis, Hemodiafiltration, Mortality, Hemodynamic Stability, Cardiovascular Diseases, Diálise Renal, Hemodiafiltração, Mortalidade, Estabilidade Hemodinâmica, Doenças Cardiovasculares

## Abstract

The desperate attempt to improve mortality, morbidity, quality of life and patient-reported outcomes in patients on hemodialysis has led to multiple attempts to improve the different modes, frequencies, and durations of dialysis sessions in the last few decades. Nothing has been more appealing than the combination of diffusion and convection in the form of hemodiafiltration. Despite the concrete evidence of better clearance of middle weight molecules and better hemodynamic stability, tangible evidence to support the universal adoption is still at a distance. Survival benefits seen in selected groups who are likely to tolerate hemodiafiltration with better vascular access and with lower comorbid burden, need to be extended to real life dialysis patients who are older than the population studied and have significantly higher comorbid burden. Technical demands of initiation hemodiafiltration, the associated costs, and the incremental benefits targeted, along with patient-reported outcomes, need to be explored further before recommending hemodiafiltration as the mode of choice.

## RESUMO

A tentativa desesperada de melhorar a mortalidade, morbidade, qualidade de vida e desfechos relatados pelos pacientes em indivíduos em hemodiálise levou a diversas tentativas de aprimorar os diferentes modos, frequências e durações das sessões de diálise nas últimas décadas. Nada foi mais atrativo do que a combinação de difusão e convecção na forma de hemodiafiltração. Apesar das evidências concretas de melhor depuração de moléculas de peso médio e melhor estabilidade hemodinâmica, evidências tangíveis para apoiar a adoção universal ainda estão distantes. Os benefícios de sobrevida observados em grupos selecionados que provavelmente toleram a hemodiafiltração com melhor acesso vascular e com menor carga de comorbidades precisam ser estendidos aos pacientes reais em diálise, que são mais velhos do que a população estudada e apresentam uma carga de comorbidades significativamente maior. As exigências técnicas do início da hemodiafiltração, os custos associados e os benefícios incrementais almejados, juntamente com os desfechos relatados pelos pacientes, precisam ser melhor explorados antes de se recomendar a hemodiafiltração como o modo de escolha.

## Introduction

Hemodialysis (HD) is still the most common mode of renal replacement therapy (RRT) worldwide, and access to health care varies among different nations according to their income category^1^. The choice of mode of RRT, an integral aspect of routine clinical care, is inlfuenced by real-world settings and nephrologists’ perspective. Shared decision making with adequate information provided to patients tends to yield greater patient satisfaction and enhance compliance^2^. New evidence is always eagerly awaited, as there is an inherent need to improve mortality, morbidity, and quality of life in patients with end stage kidney disease (ESKD). Despite the advancements made in the technology and delivery of HD, poorer outcomes are still a major concern and ways to improve are relentlessly sought. The expected life expectancy of individuals on HD remains significantly shorter compared to the general population^3^. In crude terms, survival is often worse than for breast or colon cancer^4^.

## Progression of Evidence

The initial dialysis dose, the duration, and the place of dialysis (home-based/institution-based) are explored to improve morbidity and mortality among dialysis patients. In addition, the well-known HEMO and MPO trials addressed the issue of removal of uremic toxins of higher molecular weight through high-flux hemodialysis (HF-HD). It is interesting to note that transition to high-flux (HF-HD) was driven by limited evidence in the previous decade. Initial studies did not show benefits of HF-HD in mortality or morbidity. In the HEMO study, despite achieving a high dose of dialysis and adequate clearance of β (2)-microglobulin with high-flux membrane, no difference was noted in survival, hospitalization rate, or maintenance of serum albumin levels. Benefits were seen in selected subgroups of females and patients with vintage dialysis time of more than 3.7 years, but a definite conclusion of improved survival with increased dialysis dose or use of a high-flux membrane was not achieved^5^. Again, the MPO trial, a European prospective study in HF-HD, was unconvincing and only showed a trend towards mortality benefits in *post hoc* analysis and was pronounced in specific groups of patients with low albumin and a history of diabetes^6^. Considering the increasing number of dialysis patients with diabetes and low albumin levels, the clinical relevance inferred from the MPO study led to the publication of a position statement by the European Renal Best Practice Advisory Board recommending the use of HF-HD for high-risk patients and eventually all patients due to the substantial reduction in β (2)-microglobulin levels observed in the high-flux group^7^.

Since the inception of the concept of combining diffusion and convection in the form of hemodiafiltration (HDF) in the late 1970s, it has evolved into online HDF (OL-HDF) as the standard mode of delivery with automated provision of ultrapure non-pyrogenic dialysate^8^. Although pre-dilution and mixed dilution are practiced, post-dilution HDF has been widely accepted as the common mode of delivery. Post-dilution HDF provides the best solute clearance but increase in transmembrane pressure (TMP) due to increase viscosity and clogging of membrane pores restrict the clearance, leading to fouling of the membrane. Modern online HDF machines are geared to prevent fouling through titration of the filtration fraction up to 30% and prevent excessive hemoconcentration with continuous monitoring of TMP^9^. Despite the online and real-time support in modern HDF machines, a high blood flow rate and well-functioning vascular accesses are essential for uninterrupted blood flow to achieve the recommended high-volume convection of 23L/session^9^. In practice, when the prerequisite for post-dilution HDF is not met, pre- or mixed dilution HDF are considered and also practiced more commonly in Asian countries where arteriovenous blood flow is relatively low^10^.

Middle molecular compounds are attributed to increased oxidative stress and endothelial dysfunction, leading to increased cardiovascular mortality. Increased clearance of these compounds has been associated with improved survival in observational studies and improvement in other manifestations of β (2)-microglobulin accumulation^11^. Clerance of middle molecular weight substances like beta-2 microglobulin, IL-6, TNF-alpha, p-cresol, indoxyl sulphate, and advanced glycation end products (AGE) are achievable in clinical context through HDF along with better kt/v and urea clearance^12,13,14^.

Hemodynamic stability is also better maintained in HDF compared to HF-HD^14,15^. Reduction in intradialytic hypotensive episodes driven by replenishment of substitution fluid, increase in peripheral resistance, and negative thermal balance are the probable driving force of cardiovascular benefits^16^. Reduced endothelial dysfunction associated with removal of inflammatory cytokines also contributes^12^. Unfortunately, theoretical benefits expected have not really been translated into irrefutable evidence, except for few observational studies^17,18^.

## Current Evidence

Robust evidence was sought through randomized controlled trials in the last decade, but unfortunately results often fell short for recommending HDF as the mode of choice. The Contrast study in 2012, first to compare OL-HDF with hemodialysis (low-flux hemodialysis (LF-HD) in this case) did not show any difference in all-cause mortality, but reinforced the idea of delivery of high-volume hemofiltration, as it was associated with low all-cause mortality (in the highest tertile where convective volume was >22 L/treatment and the crude mortality risk ratio was lowest at 0.62 compared to HD) in the *post hoc* convective dose analysis^19^. A Turkey study in 2012 comparing HDF and HF-HD showed no difference in all-cause mortality and non-fatal cardiovascular event rates, but a trend towards a better overall survival was noted in patients with high volume hemofiltration with a substitution volume >17.4 L^20^. Better cardiovascular outcomes and overall survival in the subgroup of patients with higher convection volume in the above studies shifted the target towards high convective volume HDF as a gold standard or standard for comparison.

In 2013, the ESHOL study group provided the propulsion for the HDF modality by showing a reduction in the primary outcome of all-cause mortality compared to conventional HF-HD. The estimated number of patients to be treated suggested that switching eight patients from hemodialysis to HDF may prevent one death per year. An added benefit of a reduction in intradialytic hypotension episodes in the HDF group was also confirmed. However, selection bias towards a healthier patient population in the HDF group by the exclusion of nearly 10% of patients due to low blood flow after randomization and lack of data regarding residual renal function could have contributed to the benefits seen. Moreover, the benefit in cardiovascular mortality remained statistically non-significant and an improvement in specific groups such as diabetic patients was not found^21^.

Pooled individual participant analysis on the effects of OL-HDF based on 4 large randomized controlled trials in 2022 indicated a trend towards a benefit in overall survival and a pronounced benefit of higher convection volumes, and the benefit extended to subgroups of patients analyzed^22^.

Overall survival benefits seen consistently with higher convective volumes in the above studies and others, which were seen extended in the *post hoc* analyses, need to be looked in deeper as these studies were not designed to avoid dose-targeting bias^23^. In the absence of a definite mechanism of mortality risk reduction, confounding factors of good dialysis access and favorable overall health status may have affected the outcome, and the benefits cannot be directly attributed to the mode of convection in HDF.

Intradialytic hypotensive episodes and myocardial stunning are associated with poorer outcomes, particularly cardiovascular in hemodialysis patients^24^. Hemodynamic stability and fewer intradialytic hypotensive episodes seen in HDF are consistent in all the above studies and in the pooled data analysis. The findings could be attributed to the cooling effect associated with HDF itself, rather than the assumed convective molecular clearance or convective clearance alone. Despite the warming of the replenishment fluid, the extracorporeal circuit temperature tends to be cooler in HDF compared to standard HD sessions, which are set at a temperature of 37°C; when temperatures of HDF and HD modalities are equilibrated, the benefit of hemodynamic stability tends to diminish^25,26^. Effects similar to those of cooling could be attributed to a reduction in the left ventricular mass and preserved ejection fraction seen in HDF patients in the long term^27^.

The well-designed and much awaited Convince study, did show a lower risk of death from any cause in patients with ESKD who were treated with high dose HDF compared to standard HF-HD. Remarkably, the recommended high dose convection volume of more than 23/L per session was achieved throughout the study in more than 90% of HDF sessions^28^. However, fewer patients than planned in the initial sample size calculation were recruited due to the COVID-19 pandemic and a lower event rate than expected (less than <10 events per 100 patient years) could have weakened the power to detect difference in both beneficial and harmful outcomes, especially when a single high dose HDF intervention is undertaken, and outcomes are likely to be confounded by many factors involved. Further, the selection of patients who are likely to tolerate high dose HDF biased the sample towards predominantly healthier patients with good vascular access, as opposed to patients commonly seen in routine clinical practice. The survival benefit seen in AVF access compared to other accesses could be due to the benefits of a good access and the recruitment of more favorable patients who are more likely to tolerate HDF. The all-cause mortality benefit seen in this study was more pronounced in patients with no history of cardiovascular disease or diabetes in the HDF arm, and the benefit was lost in presence of those conditions. Moreover, the all-cause mortality benefit seen was significantly affected by better mortality outcomes in COVID-19 infection in the HDF arm.

## Implementation of HDF

The safety profile of the provision of HDF as a modality was robust in almost all RCTs and no overt concerns were raised, given the prerequisite for hygienic and microbial standards were ensured. Structured practical approaches implemented to achieve the desired high convection volume could be adopted in different settings^28^. Further, the proposed secondary outcome of patient reported outcome measures (PROM) and cost-effectiveness need to be analyzed once data are made available to assess the impact on QALY and incremental cost-effective ratio (ICER).

Outcomes beyond mortality and cardiovascular morbidity need to be explored further, as even a single component of patient preference could determine the modality of choice. Health-related quality of life (HRQoL) and (PROM) are seldom looked into and rarely reported^29^. PROM, intended as secondary outcome of the Convince trial, and HRQoL in the H4RT trial, are likely to add the patient perspective in the choice of dialysis modality.

Additional costs and incremental benefits need to be analyzed further. Incurred cost can occur in the initial setup when establishing a water treatment unit for ultrapure water and microbiological analyses or for the purchase of additional consumables and monitors. Accurate estimation of cost effectiveness, which results from services displaced to accommodate the additional costs of the new technology, varies widely and needs to be assessed based on the existing structure^30^.

## Future

We are still a long way from emulating the widespread practice of HDF and are still not convinced of clear benefits of HDF, even though they are promising in selected groups. The outcomes of the H4RT trial, a non-blinded RCT comparing the clinical and cost-effectiveness of high-volume HDF versus high-flux HD in the treatment of ESKD would likely add evidence from real-world clinical setting to make informed choices. Updating the hemodiafiltration-pooling project with individual participant data from the present trial and from other trials would allow a more precise exploration of treatment effects across all subgroups. The impact on sustainability and the impact on ecology are also being investigated at as secondary outcome in the H4RT trial. PROM as secondary outcome in the Convince study, is also expected. More robust evidence is awaited in future rather than scattered evidence gathered through heterogenous clinical trials and evidence generation with different study methodologies.

## Conclusion

Existing conclusions are derived from heterogeneous clinical trials with different methodologies, and the benefits are largely restricted to selected subgroups of patients who are likely to tolerate HDF with good vascular access and favorable health status. Therefore, HDF is still a modality that needs to be further validated before it can be recommended as the mode of choice. Despite various trials, nephrologists remain unconvinced of its universal mortality benefit, thus inhibiting the widespread acceptance of HDF as the primary HD modality. Convincing evidence of benefits attributable of convective modality in the synergetic form of HDF is still lacking.
